# Robotic‐Assisted Electrode Array Insertion Improves Stability of Acoustic Hearing Thresholds

**DOI:** 10.1002/lary.70380

**Published:** 2026-01-21

**Authors:** Uzair A. Khan, Rachel A. Scheperle, Grant Podhajsky, Meggan J. Lind, Camille C. Dunn, Alexander D. Claussen, Bruce J. Gantz, Marlan R. Hansen

**Affiliations:** ^1^ Department of Otolaryngology—Head and Neck Surgery University of Iowa Health Care Iowa City Iowa USA; ^2^ University of Iowa Carver College of Medicine Iowa City Iowa USA

**Keywords:** cochlear implant, delayed‐onset hearing loss, hearing preservation, robotic‐assisted insertion

## Abstract

**Objective(s):**

Robotic‐assisted electrode array (EA) insertion is a promising technique that may enhance preservation of residual acoustic hearing after cochlear implant (CI) surgery. The purpose of this study is to evaluate the impact of robotic‐assisted EA insertion on rates of delayed‐onset hearing loss (DOHL).

**Methods:**

Sixty (Advanced Bionics [AB]: 30, MED‐EL: 30) adult patients underwent CI surgery with manual EA insertion and 29 (AB: 13, MED‐EL: 16) with robotic‐assisted insertion using the iotaSOFT system. The primary outcome variable was longitudinal change in low frequency pure‐tone average (LFPTA). DOHL was defined as a decrease in LFPTA of > 10 dB compared to previous best postoperative LFPTA.

**Results:**

Twenty‐two (37%) out of the 60 subjects in the manual cohort and two (7%) out of the 29 subjects in the robotic‐assisted cohort had DOHL over the entire length of available follow‐up (*p* = 0.002, Fisher's exact test, two‐tailed). When evaluating DOHL results for subjects who had LFPTA data at 12 months (±4 weeks) post initial activation, 11 (29%) out of the 38 (AB: 15, MED‐EL: 23) subjects in the manual cohort and zero (0%) out of the 18 (AB: 8, MED‐EL: 10) subjects in the robotic‐assisted cohort had DOHL (*p* = 0.011, Fisher's exact test, two‐tailed). The number needed to treat was 4.

**Conclusion:**

Robotic‐assisted EA insertion is associated with a clinically meaningful reduction in rates of DOHL. Preservation of residual acoustic hearing is a critical goal in CI surgery, and robotic‐assisted EA insertion contributes towards achieving this goal.

**Level of Evidence:**

3.

## Graphical abstract

This study evaluated the impact of robotic‐assisted electrode array insertion on rates of delayed‐onset hearing loss (DOHL). DOHL occurred in 22 of 60 subjects (37%) in the manual cohort compared with 2 of 29 subjects (7%) in the robotic‐assisted cohort, representing a statistically significant reduction (p = 0.002). By enabling a more controlled and atraumatic electrode array placement, robotic‐assisted insertion is associated with improved stability of acoustic hearing thresholds.

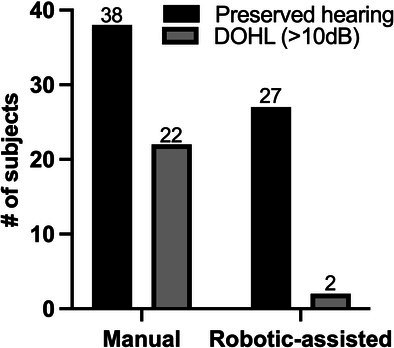

## 1 Introduction

Cochlear implants (CIs) are the established intervention for treating patients with severe‐to‐profound sensory hearing loss. Since CIs were first introduced, significant efforts to improve CI outcomes have targeted implant design, surgical planning, surgical technique, and understanding how host tissues respond to CI biomaterials [1, 2, 3, 4, 5, 6, 7, 8, 9]. Preservation of a recipient's acoustic hearing has emerged as a significant contributor to improved outcomes in CI recipients, especially with complex listening tasks such as sound quality, sound localization, hearing in noise, music appreciation, and quality of life [10, 11, 12, 13, 14, 15]. Therefore, minimizing intracochlear trauma to preserve the structure and function of the peripheral auditory system is recognized as an important goal in CI surgery.

Despite improvements to electrode array (EA) designs and surgical approaches, 50% of hearing preservation CI recipients experience loss of auditory sensitivity greater than 10 dB, and 10% to 50% lose functional residual hearing [2, 12, 16, 17, 18, 19, 20, 21]. Most patients experience a ~10–15 dB hearing loss shortly after placement of an EA, while a minority experience profound hearing loss immediately after CI that is often attributed to trauma sustained during EA insertion. Some patients with initially preserved functional acoustic thresholds, however, develop hearing loss months to years after implantation, referred to as delayed‐onset hearing loss (DOHL). DOHL is associated with an increase in electrode impedances in some, but not all instances [22, 23]. The mechanisms underlying DOHL have not been clearly elucidated. Although immediate hearing loss after CI has not been found to predict DOHL [16], it has been postulated that microscale trauma to cochlear tissues that does not cause immediate hearing loss may nevertheless trigger cochlear inflammation and tissue remodeling that ultimately contributes to DOHL. Therefore, minimizing acute insertion trauma is a critical factor in long‐term hearing preservation.

Optimizing “soft” surgical techniques for EA insertion is an important strategy to minimize acute insertion trauma and thus prevent postoperative loss of residual hearing [24, 25]. However, there are inherent limitations in human kinetics that prevent reliable and consistent reduction of acute insertion trauma during EA insertion [26, 27, 28]. Robotic‐assisted EA insertion has gained interest as a means to overcome these inherent human limitations. Robotic‐assisted EA insertion allows for a controlled and steady insertion and is associated with decreased insertion force, pressure spikes, cochlear trauma, and electrode impedances [28, 29, 30, 31, 32]. More recently, robotic‐assisted EA insertion (using two different platforms) was associated with clinically meaningful trends towards improved functional acoustic hearing preservation [31, 33]. Hearing preservation defined functionally requires a low‐frequency (125, 250, and 500 Hz) pure‐tone average (LFPTA) < 80 dB HL [34]. While these preliminary reports suggest that robotic‐assisted EA insertion is associated with improved hearing thresholds in the relative short‐term (e.g., ~12 months), the impact of robotic‐assistance on DOHL remains unknown.

The purpose of this study is to evaluate the impact of robotic‐assisted EA insertion on rates of DOHL. For this purpose, DOHL was defined more rigorously than preservation of functional acoustic hearing: a decrease in LFPTA of > 10 dB compared to previous best postoperative LFPTA—this is a metric that has been previously used to assess rates of DOHL [22]. Overall, the data demonstrate lower rates of DOHL in patients who undergo robotic‐assisted EA insertion as compared to those who undergo manual EA insertion.

## 2 Materials and Methods

### 2.1 Study Population

The study was conducted according to the guidelines for the protection of human subjects as set forth by the Institutional Review Board (IRB) at the University of Iowa. Informed consent was obtained for all procedures. Eighty‐nine adult subjects meeting Food and Drug Administration (FDA) criteria for CI were included in this study. All 89 subjects had LFPTA ≤ 70 dB HL (see Tables 1 and 2). Forty‐three (manual: 30, robotic‐assisted: 13) subjects received Advanced Bionics (AB; Table 1) CIs and 46 (manual: 30, robotic‐assisted: 16) subjects received MED‐EL CIs (Table 2).

**TABLE 1:** Characteristics of the Advanced Bionics cohort.

Variable	Manual (*n* = 30)	Robotic‐assisted (*n* = 13)
Age at surgery (mean ± SE) in years	67.6 ± 2.7	66.0 ± 5
Sex *N* (%)		
Male	16 (53.3%)	10 (76.9%)
Female	14 (46.7%)	3 (23.1%)
Etiology of hearing loss *N* (%)		
Idiopathic	13 (43%)	7 (54%)
Noise exposure	7 (23%)	2 (15%)
Familial/genetic	8 (27%)	4 (31%)
Viral	1 (3%)	0 (0%)
Iatrogenic/trauma	1 (3%)	0 (0%)
Preop LFPTA mean ± SE (range) in dB HL	49 ± 2 (25–68)	45 ± 3 (22–62)
CI array *N* (%)		
SlimJ	30 (100%)	13 (100%)

**TABLE 2:** Characteristics of the MED‐EL cohort.

Variable	Manual (*n* = 30)	Robotic‐assisted (*n* = 16)
Age at surgery (mean ± SE) in years	68.1 ± 2.8	64.4 ± 3.6
Sex *N* (%)		
Male	18 (60%)	8 (50%)
Female	12 (40%)	8 (50%)
Etiology of hearing loss *N* (%)		
Idiopathic	18 (60%)	8 (50%)
Noise exposure	6 (20%)	3 (19%)
Familial/genetic	5 (17%)	4 (25%)
Viral	1 (3%)	0 (0%)
Iatrogenic/trauma		1 (6%)
Preop LFPTA mean ± SE (range) in dB HL	46 ± 2.4 (15–70)	35 ± 3.1 (15–55)
CI array *N* (%)		
Flex 20	8 (27%)	3 (19%)
Flex 24	18 (60%)	7 (44%)
Flex 26	0 (0%)	4 (25%)
Flex 28	3 (10%)	2 (12%)
Flex Soft	1 (3%)	0 (0%)

Surgeries for the AB manual insertion group were performed between April 2018 and April 2022, and for the AB robotic‐assisted insertion group between March 2022 and February 2023. Surgeries for the MED‐EL manual insertion group were performed between May 2015 and February 2022, and for the MED‐EL robotic‐assisted insertion group between January 2021 and March 2023.

**FIGURE 1:**
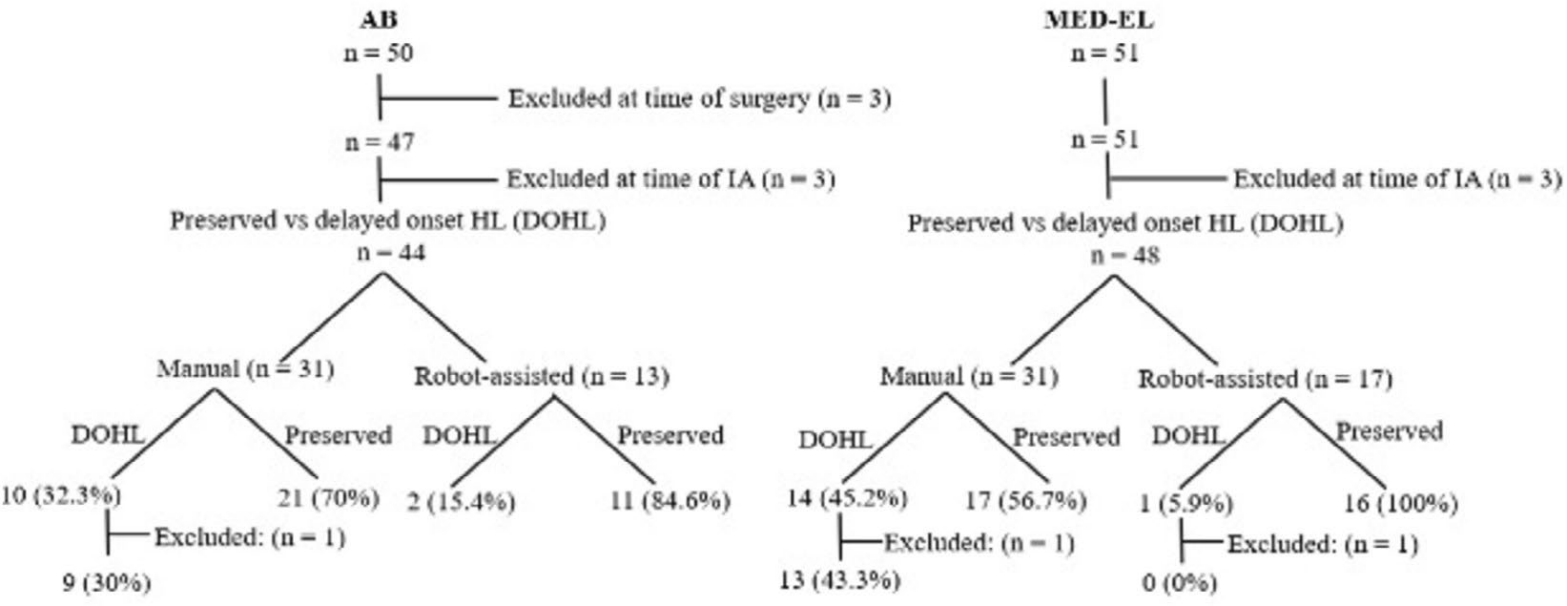
Flowchart of subject inclusion and exclusion for the Advanced Bionics (left) and MED‐EL (right) cohorts.

Figure 1 displays the flowchart for subject inclusion and exclusion for the AB and MED‐EL cohorts. The AB cohort initially had 50 subjects, of which three were excluded at the time of surgery for: (1) CSF leak that was incidentally discovered and repaired during surgery, (2) intraoperative electrode migration that was revised, and (3) concurrent tympanoplasty at the time of CI surgery. Three subjects were further excluded at initial activation (IA) for either not having any audiometric response at IA or not attending any follow‐up appointments after IA. Of the 44 remaining subjects in the AB cohort, 31 were in the manual insertion group and 13 were in the robotic‐assisted insertion group. Our final exclusion criteria involved any subjects with DOHL who also had hearing loss in the contralateral ear or no data available for the contralateral ear after IA. This excluded one additional subject from the AB‐manual‐DOHL group.

The MED‐EL cohort initially had 51 subjects, and none were excluded at time of surgery. Three subjects were excluded at IA for either not having any audiometric response at IA or not attending any follow‐up appointments after IA. Of the 48 remaining subjects in the MED‐EL cohort, 31 were in the manual insertion group and 17 were in the robotic‐assisted insertion group. Our final exclusion criteria involved any subjects with DOHL who also had hearing loss in the contralateral ear or no data available for the contralateral ear after IA. This was done to ensure that any hearing loss classified as postoperative DOHL was not attributable to symmetric, non–implant‐related etiologies. This excluded one additional subject each from the MED‐EL manual DOHL group and the MED‐EL robotic‐assisted DOHL group.

Table 1 displays the baseline characteristics of the 43 subjects (manual: 30, robotic‐assisted: 13) in the AB cohort. In the AB manual group, the mean age was 67.6 years (SE: 2.7), 53.3% were male, 46.7% were female, and the most common etiology of hearing loss was idiopathic (43%), followed by familial/genetic (27%) and noise exposure (23%). In the AB robotic‐assisted group, the average age was 66 years (SE: 5), 76.9% were male, 23.1% were female, and the most common etiology of hearing loss was idiopathic (54%), followed by familial/genetic (31%) and noise exposure (15%). The mean preoperative LFPTA in the AB manual group was 49 (SE: 2, range: 25–68) dB compared to 45 (SE: 3, range 22–62) dB in the AB robotic‐assisted group (Welch's *t*‐test, *p* = 0.35). All 43 subjects in the AB cohort received a SlimJ EA.

Table 2 displays the baseline characteristics of the 46 subjects (manual: 30, robotic‐assisted: 16) in the MED‐EL cohort. In the MED‐EL manual group, the mean age was 68.1 years (SE: 2.8), 60% were male, 40% were female, and the most common etiology of hearing loss was idiopathic (60%), followed by noise exposure (20%) and familial/genetic (17%). The EAs used in the MED‐EL manual group were Flex20 (27%), Flex24 (60%), Flex 28 (10%), and Flex Soft (3%). In the MED‐EL robotic‐assisted group, the mean age was 64.4 years (SE: 3.6), 50% were male, 50% were female, and the most common etiology of hearing loss was idiopathic (50%), followed by familial/genetic (25%) and noise exposure (19%). The mean preoperative LFPTA for the MED‐EL manual group was 46 (SE: 2.4, range: 15–70) dB compared to 35 (SE: 3.1, range: 15–55) dB in the MED‐EL robotic‐assisted group (Welch's *t*‐test, *p* = 0.01). The EAs used in the MED‐EL robotic‐assisted group were Flex20 (19%), Flex24 (44%), Flex26 (25%), and Flex 28 (12%).

### 2.2 Manual Insertion

All CI procedures involving manual insertion were performed by three faculty neurotologists at a single institution who routinely employ soft surgical techniques. The procedure involved a standard postauricular approach with mastoidectomy and posterior tympanotomy to gain access to the round window. For all insertions in this study, the EA was advanced manually into the cochlea through the round window. Steroids were routinely administered at multiple time points with different modes of delivery. All patients received 10 mg intravenous dexamethasone during the surgery. Dexamethasone was applied locally through the facial recess to bathe the round window niche prior to EA insertion. And finally, oral steroids (prednisone) were prescribed postoperatively. Over the time period of this study, the use of preoperative image analysis to influence EA choice and the inclusion of intraoperative electrocochleography (ECochG) monitoring during EA insertion were under investigation and clinical implementation. They were gradually adopted into standard clinical practice, being used in both groups as soon as they were available.

### 2.3 Robotic‐Assisted Insertion

As above, all CI procedures involving robotic‐assisted insertion were performed by the same three faculty neurotologists at the same institution.

All robotic‐assisted surgeries used the iotaSOFT system (iotaMotion Inc., Iowa City, IA), a single‐use robotic‐assisted EA insertion system as previously described [20, 33]. Similar to the manual insertion group, the procedure involved a standard postauricular incision, mastoidectomy and posterior tympanotomy to gain access to the round window. The iotaSOFT system was then used to advance the EA near the round window. Following the opening of the round window membrane, the EA was advanced into the cochlea at 0.2 mm/s using the iotaSOFT system. This system can advance, retract, stop or reverse at speeds between 0.1 and 1 at 0.1 mm/s intervals. At our institution, the standard intracochlear insertion speed is 0.2 mm/s. Administration of steroids was similar to the manual insertion group as described above.

### 2.4 Audiometric Data and Follow‐Up

The primary outcome variable was the longitudinal change in the LFPTA, defined as mean audiometric threshold at 125, 250, and 500 Hz [34]. Subjects had behavioral audiometric thresholds measured preoperatively, at IA (typically, within 4 weeks of surgery), and at subsequent programming visits (after IA at 2 weeks, 1 month, 3 months, 6 months, 12 months, and yearly after that). The visit schedule was occasionally adjusted to accommodate patient availability/preferences or as clinically indicated. LFPTA was used to evaluate DOHL, defined as a decrease in LFPTA of > 10 dB compared to previous best postoperative LFPTA [22]. A transient change > 10 dB was not counted as DOHL.

The group analysis was first performed using all available timepoints for each subject. However, the length of follow‐up differed across groups. The average length of follow up in the AB manual group and AB robotic‐assisted group was 125 weeks and 58 weeks, respectively. The average length of follow up in the MED‐EL manual group and MED‐EL robotic‐assisted group was 164 weeks and 44 weeks, respectively. Differences in average follow‐up between the manual and robotic‐assisted groups reflect the later introduction of robotic‐assisted technique during the study timeline. To address these differences, the rates of DOHL were also analyzed using only the subset of subjects who had follow‐up data at 1 year (±4 weeks) post IA. This criterion resulted in the exclusion of 15 AB manual, 5 AB robotic‐assisted, 7 MED‐EL manual, and 6 MED‐EL robotic‐assisted subjects. Thus 56 subjects were included in the 1‐year post‐IA DOHL analysis.

## 3 Results

### 3.1 DOHL Across the Study Period

**FIGURE 2:**
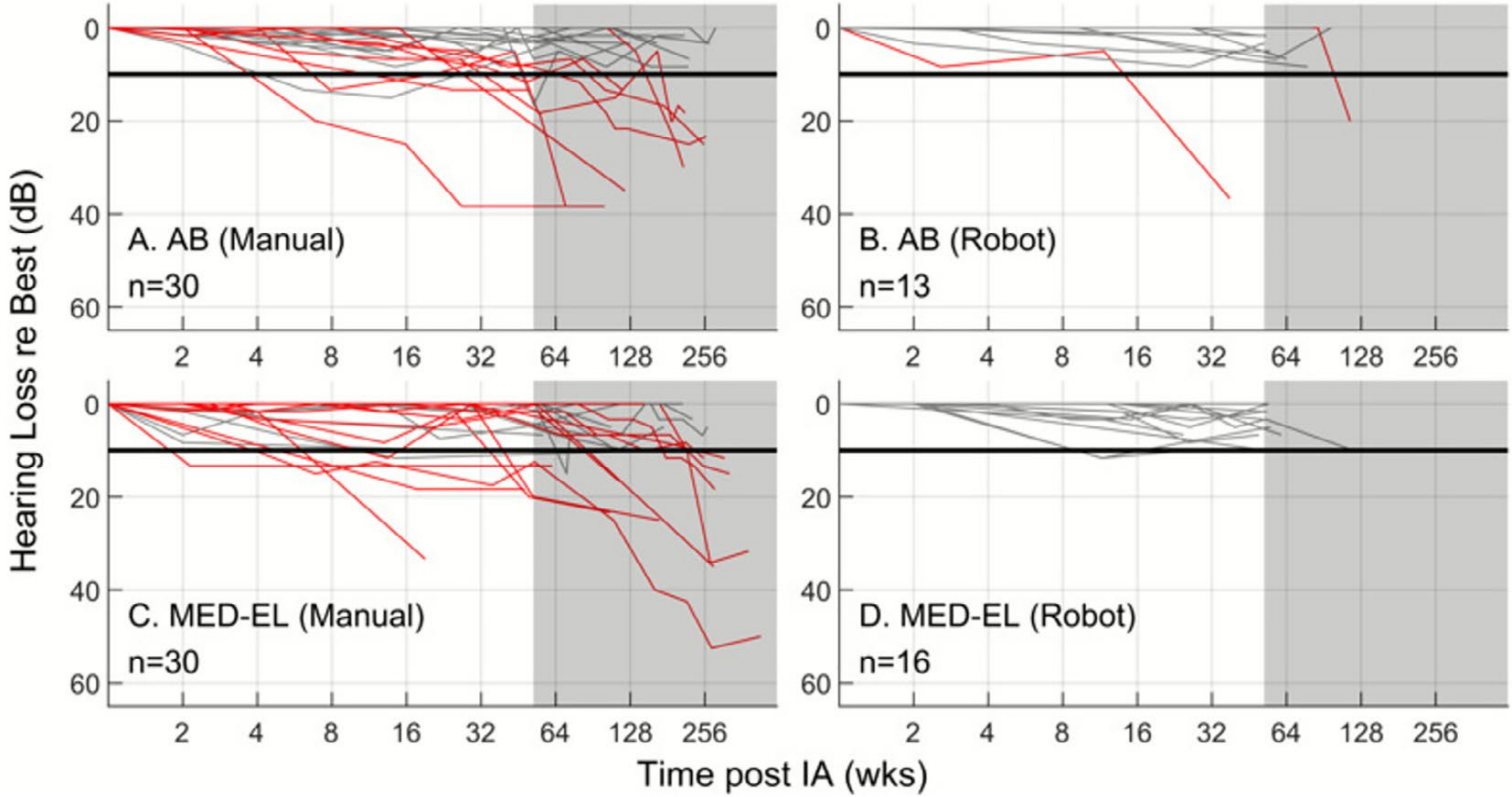
Change in LFPTA from previous best (each datapoint) for individual subjects (no DOHL: gray line, DOHL: red line) across available time in the (A) Advanced Bionics (AB) manual, (B) AB robotic‐assisted, (C) MED‐EL manual, and (D) MED‐EL robotic‐assisted groups. Gray region represents time after 52 weeks. [Color figure can be viewed in the online issue, which is available at www.laryngoscope.com]

**FIGURE 3:**
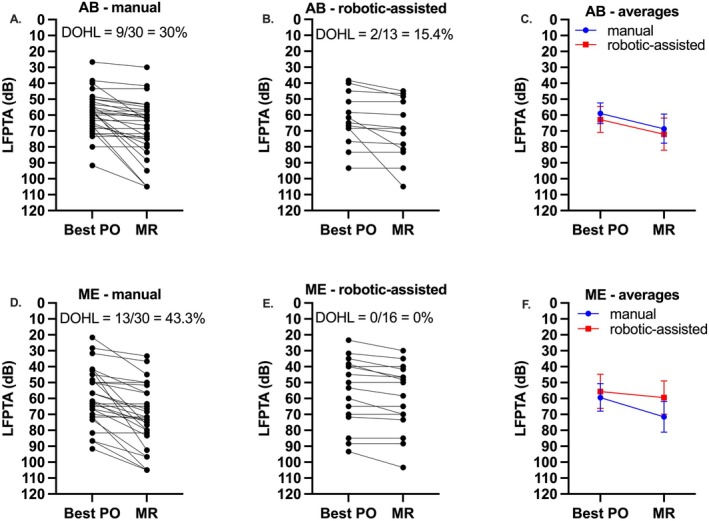
Absolute change in LFPTA for each individual subject (line) between best postoperative (Best PO) and most recent (MR) timepoints for the (A) Advanced Bionics (AB) manual, (B) AB robotic‐assisted, (D) MED‐EL (ME) manual, and (E) ME robotic‐assisted groups. Averages across subjects for AB (C, manual: blue, robotic‐assisted: red) and ME groups (F, manual: blue, robotic‐assisted: red) are displayed. Error bars reflect standard deviation. [Color figure can be viewed in the online issue, which is available at www.laryngoscope.com]

To explore long‐term hearing stability after CI, we used as rigorous a criterium as feasible (> 10 dB compared to the best postoperative LFPTA). In our experience, hearing changes at IA are comprised of both sensorineural and conductive components that can eventually resolve. Therefore, we used the best postoperative LFPTA to help mitigate any contribution of temporary conductive component. There was no statistically significant difference in overall LFPTA at IA (*p* = 0.98, Welch's *t*‐test) between the manual (65.4 dB, *n* = 60) and robotic‐assisted (65.5 dB, *n* = 29) cohorts. Figure 2 displays the changes in LFPTA as compared to the previous best LFPTA for the entire duration of follow‐up. Each line represents an individual subject. The color red is used for individuals with DOHL. When considering all time points available for each subject, the AB manual group had 9/30 (30%) subjects with DOHL compared to 2/13 (15.4%) subjects in the AB robotic‐assisted group (*p* = 0.4559, Fisher's exact test, two‐tailed). The MED‐EL manual group had 13/30 (43.3%) subjects with DOHL compared to 0/16 (0%) subjects in the MED‐EL robotic‐assisted group (*p* = 0.0015, Fisher's exact test, two‐tailed). These differences are also illustrated in Figure 3, which displays the changes in LFPTA for each individual subject between their best postoperative LFPTA and their most recent LFPTA.

**FIGURE 4:**
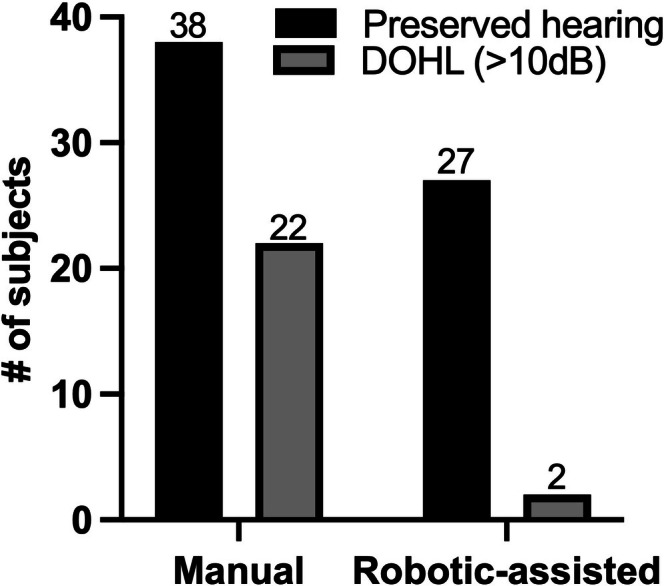
Rates of delayed onset of hearing loss across the duration of the study period. 22 (AB: 9, MED‐EL: 13) out of the 60 (AB: 30, MED‐EL: 30) subjects in the manual cohort and two (AB: 2, MED‐EL: 0) out of the 29 (AB: 13, MED‐EL: 16) subjects in the robotic‐assisted cohort had DOHL over the entire length of available follow‐up.

As displayed in Figure 4, when combining the data from AB and MED‐EL cohorts, the manual group had 22/60 (36.7%) of subjects with DOHL compared to 2/29 (6.9%) subjects in the robotic‐assisted group (*p* = 0.0024, Fisher's exact test, two‐tailed).

### 3.2 DOHL—1‐Year Comparison

**FIGURE 5:**
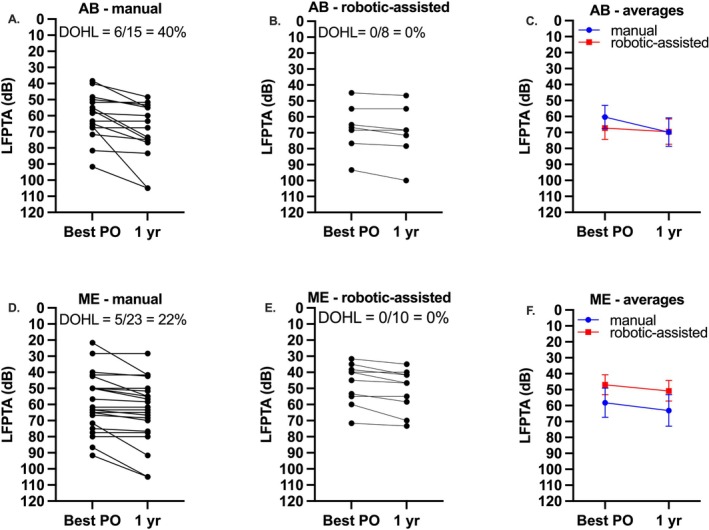
Absolute change in LFPTA for each individual subject (line) between best postoperative prior to 1 year (1 year) (Best PO) and 1 year timepoints for the (A) Advanced Bionics (AB) manual, (B) AB robotic‐assisted, (D) MED‐EL (ME) manual, and (E) ME robotic‐assisted groups. Averages across subjects for AB (C, manual: Blue, robotic‐assisted: Red) and ME groups (F, manual: blue, robotic‐assisted: red) are displayed—error bars reflect standard deviation. [Color figure can be viewed in the online issue, which is available at www.laryngoscope.com]

**FIGURE 6:**
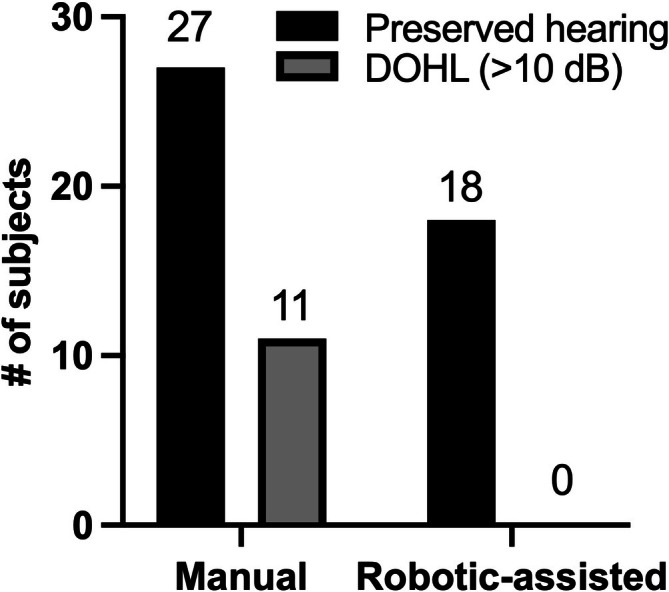
Rates of delayed onset of hearing loss at 1 year postoperatively. 11 (AB: 6, MED‐EL: 5) out of the 38 (AB: 15, MED‐EL: 23) subjects in the manual cohort and zero (AB: 0, MED‐EL: 0) out of the 18 (AB: 8, MED‐EL: 10) subjects in the robotic‐assisted cohort had DOHL when only comparing subjects who had data points at 1 year post IA. If the two subjects with DOHL before 1 year but who have not yet been followed for a full 12 months are included, these numbers adjust to 12 (manual DOHL) and 1 (robotic‐assisted DOHL).

As the time since implantation for the manually inserted group is significantly longer than the robotic‐assisted group, we also explored LFPTA threshold changes at the same timepoint (52 weeks postoperatively). The gray region in Figure 2 marks 52 weeks post IA for reference. Figures 5 and 6 display DOHL results for the subset of subjects who had LFPTA data at 12 months (±4 weeks) post IA (note: one subject from each of the AB control and MED‐EL control groups with an LFPTA of 105 both before and after the missed 12‐months ±4 weeks time point was included). The AB manual group had 6/15 (40%) subjects with DOHL compared to 0/8 (0%) subjects in the AB robotic‐assisted group (*p* = 0.058, Fisher's exact test, two‐tailed). The MED‐EL manual group had 5/23 (22%) subjects with DOHL compared to 0/10 (0%) subjects in the MED‐EL robotic‐assisted group (*p* = 0.291, Fisher's exact test, two‐tailed). As displayed in Figure 6, when combining the data from AB and MED‐EL cohorts to increase the sample size and statistical power, the combined manual group had 11/38 (29%) subjects with DOHL compared to 0/18 (0%) subjects in the combined robotic‐assisted group (*p* = 0.0108, Fisher's exact test, two‐tailed).

As shown in Figure 2, one subject in the robotic‐assisted AB group experienced a change in LFPTA of greater than 10 dB (~37 dB) but was not included in the one‐year analysis shown in Figure 6. This is because that subject did not meet the criteria for the one‐year analysis, which required a follow‐up at 1 year ±4 weeks. Similarly, one subject in the MED‐EL control group had a change in LFPTA of greater than 10 dB (~33 dB) but was also excluded from the one‐year analysis for the same reason. Including these two subjects does not change the statistical significance of the results.

### 3.3 Number Needed to Treat (NNT)

The NNT is a quantitative measure defined as the number of subjects that need to be treated with a specific intervention for one person to benefit from it compared to control subjects. It is calculated by dividing 1 by the absolute risk difference (ARD).

When evaluating the entire length of follow up, the ARD for DOHL within the AB (manual—robotic‐assisted), MED‐EL (manual—robotic‐assisted), and combined AB + MED‐EL (manual—robotic‐assisted) groups was 0.15 (0.30–0.15), 0.43 (0.43–0), and 0.3 (0.37–0.07), respectively. Consequently, the NNT with robotic‐assisted EA insertion to prevent one additional negative outcome of DOHL would be 7 (1/0.15), 3 (1/0.43), and 4 (1/0.3) for the AB, MED‐EL, and combined AB + MED‐EL groups, respectively.

When evaluating DOHL results for subjects who had LFPTA data at 12 months (±4 weeks) post IA, the ARD for DOHL within the AB (manual—robotic‐assisted), MED‐EL (manual—robotic‐assisted), and combined AB + MED‐EL (manual—robotic‐assisted) groups was 0.40 (0.40–0), 0.22 (0.22–0), and 0.29 (0.29–0), respectively. Consequently, the NNT with robotic‐assisted EA insertion to prevent one additional negative outcome of DOHL would be 3 (1/0.40), 5 (1/0.22), and 4 (1/0.29) for the AB, MED‐EL, and combined AB + MED‐EL groups, respectively.

## 4 Discussion

Robotic‐assisted EA insertion was associated with a clinically meaningful reduction in rates of DOHL as compared to manual insertion. When evaluating the entire length of follow up, the differences in rates of DOHL between robotic‐assisted and manual insertion for the AB, MED‐EL, and combined groups were 15% (manual: 30%, robotic‐assisted: 15%, *p* = 0.46), 43% (manual: 43%, robotic‐assisted: 0%, *p* = 0.002) and 30% (manual: 37%, robotic‐assisted: 7%, *p* = 0.002), respectively. When evaluating subjects who had follow up at the 1 year time point, the differences in rates of DOHL between robotic‐assisted and manual insertion for the AB, MED‐EL, and combined groups were 40% (manual: 40%, robotic‐assisted: 0%, *p* = 0.06), 22% (manual: 22%, robotic‐assisted: 0%, *p* = 0.29) and 29% (manual: 29%, robotic‐assisted: 0%, *p* = 0.011), respectively. To highlight, when all data are combined (AB + MED‐EL), the difference in rates of DOHL between robotic‐assisted EA insertion and manual insertion is around 30% for the entire length of follow up (30%, *p* = 0.002) and at 1 year (29%, *p* = 0.011). This is a more drastic difference in outcomes as compared to our previous report that evaluated functional acoustic hearing (< 80 dB HL), where the difference in hearing preservation rate between manual and robotic‐assisted insertion was 14% [33].

The NNT is a quantitative measure used to evaluate the clinical value of an intervention, defined as the number of subjects that need to be treated with a particular intervention (robotic‐assisted EA insertion) for one person to benefit (prevent DOHL) as compared to control (manual insertion) subjects. The NNT when looking at the combined (AB + MED‐EL) data is 4, at both the 1‐year time point and entire length of follow up. An NNT of 4 reflects an effective intervention, underscoring the clinical value of robotic‐assisted EA insertion. Of note, although the exclusions described in Figure 1 were not included in any analysis, the NNT of 4 would remain unchanged if the exclusions made at IA due to absent audiometric thresholds, or those made after determination of DOHL due to hearing loss in the contralateral ear or unavailable contralateral ear data, were factored in. These data are largely in line with the recently published *n* = 7 NNT calculation based on overall hearing preservation levels comparing manual and robotic‐assisted EA insertion [33].

The effectiveness of robotic‐assisted EA insertion in reducing DOHL may be attributed to its capacity to overcome the inherent limitations of human kinetics and reduce acute insertion trauma via slower and steadier insertions, decreased insertion forces, and reduced spikes in intracochlear pressure [28, 29, 30, 31, 32]. Although acute insertional trauma is generally associated with immediate hearing loss after CI, and immediate hearing loss has not been found to predict DOHL [16], acute insertional trauma may nonetheless trigger biological processes that ultimately contribute to DOHL. Still, further research is required to elucidate the mechanisms underlying DOHL and how robotic‐assisted EA insertion reduces its incidence.

Limitations of this study include retrospective design. Although data were collected prospectively, future research should involve a prospective, randomized trial. A randomized controlled trial will also help investigate whether the effectiveness of robotic‐assisted EA insertion varies between different types of EAs/manufacturers; currently there is not sufficient evidence to make such a conclusion. Additionally, we only evaluated one commercially available motorized insertion system. Although the benefits of robotic‐assisted EA insertion should apply to other systems that allow for slow and steady EA insertion, the effectiveness of other systems should be evaluated separately [35, 36].

Preservation of acoustic hearing improves outcomes in CI recipients, particularly in challenging listening situations, and is therefore a critical goal in CI surgery. Accordingly, every effort should be made to preserve residual acoustic hearing whether by improving CI design, surgical planning, or surgical technique. Robotic‐assisted EA insertion enhances surgical technique by enabling a controlled, slow, and steady insertion. The degree of control facilitated by these systems surpasses the abilities of human motor control. Such fine control reduces acute insertional trauma to the delicate cochlear structures during EA insertion and, as demonstrated by our results, aids in long‐term preservation of cochlear function.

## 5 Conclusion

Robotic‐assisted EA insertion is associated with a clinically meaningful reduction in rates of DOHL. Preservation of residual acoustic hearing improves outcomes in CI recipients, particularly in challenging listening situations, and is therefore a critical goal in CI surgery. By facilitating a more atraumatic and controlled EA placement, robotic‐assisted EA insertion contributes meaningfully towards achieving this goal.

## Funding

This work was supported by NIH, National Institute on Deafness and Other Communication Disorders, P50‐DC000242 and T32‐DC000040.

## Conflicts of Interest

C.C.D. serves as a consultant for Cochlear Corporation and iotaMotion Inc. R.A.S. serves as a consultant for iotaMotion Inc. B.J.G. serves as a consultant for Cochlear Corporation and iotaMotion Inc. M.R.H. is the co‐founder and chief medical officer of iotaMotion Inc. with equity interest. The other authors declare no conflicts of interest.

## Data Availability

The data that support the findings of this study are available on request from the corresponding author. The data are not publicly available due to privacy or ethical restrictions.
